# Local release of tacrolimus from hydrogel-based drug delivery system is controlled by inflammatory enzymes *in vivo* and can be monitored non-invasively using *in vivo* imaging

**DOI:** 10.1371/journal.pone.0203409

**Published:** 2018-08-30

**Authors:** Dzhuliya Dzhonova, Radu Olariu, Jonathan Leckenby, Ashish Dhayani, Praveen Kumar Vemula, Jean-Christophe Prost, Yara Banz, Adriano Taddeo, Robert Rieben

**Affiliations:** 1 Department for BioMedical Research (DBMR), University of Bern, Bern, Switzerland; 2 Graduate School for Cellular and Biomedical Sciences, University of Bern, Bern, Switzerland; 3 Department of Plastic and Hand Surgery, Inselspital, Bern University Hospital, Bern, Switzerland; 4 Institute for Stem Cell Biology and Regenerative Medicine, Bangalore, India; 5 The School of Chemical and Biotechnology, SASTRA University, Tamil Nadu, India; 6 Center of Laboratory Medicine, University Institute of Clinical Chemistry, University Hospital, Switzerland; 7 Institute of Pathology, University of Bern, Bern, Switzerland; University of Missouri Columbia, UNITED STATES

## Abstract

**Background:**

Local drug delivery systems that adjust the release of immunosuppressive drug in response to the nature and intensity of inflammation represent a promising approach to reduce systemic immunosuppression and its side effects in allotransplantation. Here we aimed to demonstrate that release of tacrolimus from triglycerol monostearate hydrogel is inflammation-dependent *in vivo*. We further report that by loading the hydrogel with a near-infrared dye, it is possible to monitor drug release non-invasively in an *in vivo* model of vascularized composite allotransplantation.

**Materials and methods:**

Inflammation was induced by local challenge with lipopolysaccharides in naïve rats 7 days after injection of tacrolimus-loaded hydrogel in the hind limb. Tacrolimus levels in blood and tissues were measured at selected time points. A near-infrared dye was encapsulated in the hydrogel together with tacrolimus in order to monitor hydrogel deposits and drug release *in vitro* and *in vivo* in a model of vascularized composite allotransplantation.

**Results:**

Injection of lipopolysaccharides led to increased blood and skin tacrolimus levels (p = 0.0076, day 7 vs. day 12 in blood, and p = 0.0007 in treated limbs, 48 h after injection compared to controls). Moreover, lipopolysaccharides-injected animals had higher tacrolimus levels in treated limbs compared to contralateral limbs (p = 0.0003 for skin and p = 0.0053 for muscle). Imaging of hydrogel deposits and tacrolimus release was achieved by encapsulating near-infrared dye in the hydrogel for 160 days. The correlation of tacrolimus and near-infrared dye release from hydrogel was R^2^ = 0.6297 and R^2^ = 0.5619 in blood and grafts of transplanted animals respectively and R^2^ = 0.6066 *in vitro*.

**Conclusions:**

Here we demonstrate the inflammation-responsiveness of a tacrolimus-loaded hydrogel *in vivo*. Moreover, we show that encapsulating a near-infrared dye in the hydrogel provides a reliable correlation of tacrolimus and dye release from the hydrogel, and an accessible non-invasive method for monitoring drug release from hydrogel deposits.

## Introduction

Vascularized composite allotransplantation (VCA)–the transplantation of tissues, such as hands, face and abdominal wall–has experienced an exhilarating development in the past two decades. At present 69 hand transplant recipients (40 bilateral and 29 unilateral) have been registered in the International Registry on Hand and Composite Tissue Transplantation (https://www.handregistry.com), most of whom demonstrate excellent survival, function and appearance [1]. The recipients are typically young and healthy people who need life-long immunosuppression to prevent rejection in their grafts. Unfortunately, the use of systemic immunosuppression is associated with increased risks of infections, metabolic disturbances, and cancer. As recently reported, 32.3% of hand transplant recipients experienced an opportunistic bacterial infection, 41.5% had hyperglycemia and two of the 66 patients included in the report developed malignancies [1].

Unlike other organ transplantations, VCA are readily available for monitoring and drug delivery. Therefore, several groups have developed drug delivery approaches for targeted immunosuppression, with the aim to decrease systemic toxicity and improve graft outcome. These approaches have been recently reviewed [2] and include the use of topical tacrolimus (TAC) [3,4] and clobetasol [4], intra-graft injections of TAC [5] and biodegradable disks containing TAC-loaded microspheres [6]. We have been developing a novel drug delivery system for localized immunosuppression aimed at reducing systemic immunosuppression-related adverse effects by decreasing the total drug uptake required to prevent rejection. The system is composed of a triglycerol monostearate (TGMS) hydrogel loaded with the immunosuppressive TAC. TGMS hydrogel loaded with TAC (TGMS-TAC) is injectable subcutaneously into the VCA graft. We previously demonstrated that the hydrogel releases the encapsulated TAC in response to elevated levels of inflammation-related enzymes *in vitro* [7]. However, the dependency of TAC release on an inflammatory reaction has not yet been demonstrated *in vivo*. Moreover, we reported that repeated intra-graft TGMS-TAC injections in a Brown Norway-to-Lewis rat hind-limb transplantation model prolonged graft survival for >280 days, with sub-therapeutic drug levels in blood for extended periods of time [8], making this approach a promising alternative to systemic immunosuppression in VCA patients. Although targeted immunosuppression has the potential to increase therapeutic index, currently only the topical application of TAC is used in the clinic to treat rejection in VCA patients [9]. Notably, intra-graft levels of immunosuppressive drugs measured in biopsies have been described to be more accurate markers of immunosuppression than trough levels [10], and could be particularly critical in the case of local immunosuppression. Nevertheless, frequent tissue biopsy collection for drug monitoring is an unpractical, painful and feared procedure. A potential solution could be the incorporation of a surrogate marker that can be non-invasively detected and provide information on the availability of TAC in the hydrogel deposit as well as in the surrounding tissue.

Near-infra-red dyes (NIRD) are used for *in vivo* imaging of hydrogels [11], making them attractive candidates for visualizing drug delivery systems for local immunosuppression. NIRD are safe for administration in the body, emit light at wavelengths in which tissue autofluorescence is low, and can be detected by common instruments for fluorescence imaging. NIRD have proven to be effective for imaging of sentinel lymph nodes [12] and cancer [13], with a penetration depth in various tissues of up to several centimeters [14].

Here, we aimed to demonstrate the inflammation-responsiveness of TAC release from TGMS-TAC *in vivo*, in naïve rats treated with TGMS-TAC and challenged with lipopolysaccharides (LPS) near to the hydrogel deposits. We compared blood and tissue TAC levels at selected time points to a control unchallenged group in order to understand whether LPS injection could lead to an increase in TAC release from the hydrogel. In addition, we examined the hydrogel deposits for foreign-body reaction. Furthermore we hypothesized that NIRD encapsulated in TGMS-TAC would allow for non-invasive monitoring of the hydrogel deposits and TAC release in a VCA model, which could be used a surrogate marker of tacrolimus release. To this aim, we incorporated NIRD in TGMS-TAC (TGMS-TAC-NIRD) and compared the TAC and NIRD release from the hydrogel *in vitro* and *in vivo*. Moreover, to understand whether NIRD can be used to visualize hydrogel deposits and drug release in grafted tissues after VCA, Brown Norway-to-Lewis hind limb transplantations were performed and TGMS-TAC-NIRD hydrogel deposits were visualized in a long-term *in vivo* experiment.

## Materials and methods

### Animals

Male Brown Norway and Lewis rats (6–8 weeks old weighing 200 to 250 g) were purchased from Charles Rivers Breeding Laboratories, Germany. Animals were kept in specific pathogen-free conditions. Experiments were planned, carried out and reported in agreement with current 3R and ARRIVE guidelines [15] and approved according to Swiss animal protection laws by the Veterinary Authorities of the Canton Bern, Switzerland (license no. BE94/15). Sample size was calculated to be n = 6 per group, with G*Power 3 software [16], with the following inputs:

t tests—Means: Difference between two independent means (two groups); Analysis: A priori: Compute required sample size;

Input: Tail(s) = Two; Effect size d = 2; α error probability = 0.05; Power (1-β error probability) = 0.85; Allocation ratio N2/N1 = 1.

The number of animals that were treated with LPS and kept for 50 days for blood measurements were reduced to n = 3, after post-hoc analyses revealed sufficient power. This was done to minimize the number of animals potentially suffering for extended periods of time. Post-hoc power analyses calculated Power (1-β error probability) = 0.89, with G*Power 3 software, using the following inputs:

t tests—Means: Difference between two independent means (two groups); Analysis: Post-hoc: Compute achieved power–given α, sample size, and effect size;

Input: Tail(s) = Two; Effect size d = 2.64033; α error probability = 0.05; Sample size group 1 = 6; Sample size group 2 = 3.

### TGMS-TAC preparation

Triglycerol monostearate (TGMS) was purchased from AK Scientific and used for all release kinetics studies and in vivo studies. Lipase (L0777) from Thermomyces lanuginosus was procured from Sigma Aldrich. IRDye 800CW carboxylate (P/N 929–09406) was obtained from LI-COR Biosciences. Snakeskin dialysis tubing (22mm, Cat # 68035) with a molecular weight cut off of 3.5kDa was used from Thermo Scientific and was used for release kinetics experiments.

In order to prepare the hydrogel, 10% (w/v) of TGMS was weighed in a glass vial along with 7mg/ml of tacrolimus and dissolved in 20% (v/v) DMSO by heating up to 60–70°C. The mixture was allowed to come to room temperature and then re-heated with the addition of IR dye 100μg/ml and water. The whole mixture was taken in 1ml syringes and allowed to come to room temperature slowly. Gelation is said to have occurred when there is no gravitational flow upon movement or inversion. In order to decide upon drug concentration, encapsulation of various amounts of TAC (from 2–15 mg/ml) was systemically studied. Stable gel formation was observed by encapsulating maximum of 10 mg/ml of TAC in TGMS gels. However, to minimize the initial burst release, 7 mg/ml of TAC gels were used for further experiments. Similarly, 50 to 500 μg of NIRD has been encapsulated in these stable TGMS gels. However, at 100 μg/ml concentration NIRD gets stable encapsulation and does not have non-specific release. Thus, TGMS-TAC-NIRD were optimized and used at final amounts of 7mg/ml tacrolimus and 100 μg/ml NIRD respectively and used for further studies.

Limulus amebocyte lysate test (Pyrogent 03 Plus, Lonza Group, Basel, Switzerland) was used for pyrogen detection according to the manufacturer’s instructions and TGMS-TAC and TGMS-TAC-NIRD were considered pyrogen-free if a 1:10 dilution of hydrogel in sterile water resulted in a negative test result.

### TAC release in response to local inflammatory stimulus *in vivo*

Naïve Lewis rats received 1 mL TGMS-TAC loaded with 7 mg TAC subcutaneously in the graft. Four deposits of TGMS-TAC of 250 μL each were injected in the zones of biceps femoris, gastrocnemius, tibialis anterior, and vastus muscles. Animals were randomly assigned to two groups–untreated control (n = 12), and experimental group, the latter receiving a subcutaneous injection of 100 μg LPS (Lipopolysaccharides from *E*.*coli* O111:B4, γ**-**irradiated, BioXtra, Sigma) dissolved in 100 μL phosphate buffered saline (PBS) near the gel deposits (n = 9). In 6 of the control and 3 of the LPS-treated animals peripheral blood was collected from the sublingual vein in EDTA coated tubes (Sarstedt, Nümbrecht, Germany) and stored at -20° C until use. TAC concentrations in blood were assessed using the Kit MS1100 (ClinMass Complete Kit, advanced, for Immunosuppressants in Whole Blood, RECIPE Chemicals + Instruments GmbH, Munich, Germany) and quantified by LC-MS/MS. Blood TAC levels were measured for 50 days. The remaining 6 animals per group were sacrificed 9 days after TGMS-TAC injection (48 h after LPS challenge) and skin, muscle and fat pad from the treated and contralateral untreated hind limbs were snap frozen and stored at -20° C for TAC measurements. At least one hydrogel deposit per animal was formalin fixed, paraffin embedded, and 5μm thick sections were stained with Hematoxylin and Eosin and submitted to a blinded pathologist for evaluation. Representative pictures were taken with Pannoramic Scanner (panoramic 250, 3DHISTECH Ltd., Budapest, Hungary), using dedicated software Pannoramic Viewer (3DHISTECH Ltd.).

### Hind limb transplantation and treatment

Hind limb transplantations were performed using a two-surgeon method as previously described [5]. The transplanted rats received 1 ml TGMS-TAC-NIRD subcutaneously in the graft (n = 5). Four deposits of TGMS-TAC-NIRD of 250 μL each were injected in the zones of biceps femoris, gastrocnemius, tibialis anterior, and vastus muscles. Animals were inspected on a daily basis for weight loss and signs of pain/distress [17] or rejection. Rejection was macroscopically determined as grade 0 – none; 1 – edema, erythema; 2 – epidermolysis, desquamation; 3 – frank necrosis and mummification. Near-infrared imaging of gel deposits, as well as NIRD and TAC measurements in graft and plasma, were performed at selected time points and compared.

### Near-infrared dye analyses

Blood: Peripheral blood was collected from the sublingual vein in EDTA coated tubes (Sarstedt) and centrifuged at 1500 rpm for 10 minutes. Plasma was collected, placed in Corning 96 well black polystyrene microplates (clear flat bottom, black polystyrene, matrix active group TC-treated, Sigma) and immediately imaged with a LI-COR Odyssey instrument (LI-COR Biosciences) at 800 nm. Imaging conditions: Laser – 800nm; Intensity – L2.0 (minimum), resolution – 169μm, quality – medium, focus offset – 4mm (maximum), identical brightness and contrast. Naïve Lewis rat blood plasma was used for subtraction of background fluorescence.

Graft: TGMS-TAC-NIRD deposits were imaged *in situ* in live animals using LI-COR Odyssey instrument (LI-COR Biosciences) at 800 nm. Imaging conditions were identical to blood imaging. Animals were kept under anesthesia: 1–1.5% Isoflurane (AbbVie AG, North Chicago, IL, United States) with 0.6 L/min oxygen, to prevent limb movement during image acquisition. Hind limbs were shaved prior to imaging for consistency. Three native Lewis hind limbs were shaved and imaged prior transplantation and TGMS-TAC-NIRD injection. The mean value was used for subtraction of background fluorescence.

Blood and graft infrared dye signal at 800 nm was measured in entire plate wells and imaged limbs. Overexposed areas were excluded. To normalize the signal to the size of area and to avoid differences in signal distribution across area, an arbitrary area size was chosen; the average signal for such selection was calculated, and used for further analyses.

### Tacrolimus analyses

Blood: Peripheral blood was collected, stored and quantified as previously described.

Tissue: Skin, muscle and fat bad biopsies from treated, untreated, and contralateral limbs were excised, weighed, snap frozen, and stored at -20° C until use.

The sample preparation for LC-MS/MS was performed using the MS1312 kit from Recipe as internal standard. TAC and internal standard were dissolved in 70% (v/v) methanol solution in water. Standard spiking solution was prepared to build up a calibration curve between 0.3 and 65 ng/mL. The frozen tissues were gently thawed at room temperature. For blank matrix, samples without TAC treatment were used. A blank matrix was prepared by adding 1000 μL of precipitation solution to untreated tissue. A volume of 40 μL of internal standard solution and 960 μL of precipitation solution were added to the treated samples. All samples were then ground with five stainless steel balls for 30 minutes at 25 Hz. The tubes were centrifuged 5 minutes at 4° C and 20’000 g. 500 μL of the tissue extract was filtered with a Mini-Uni Prep G2 vials (GE Healthcare, Chicago, USA).

Chromatographic analyses were performed on an Acquity I-Class system (Waters, Milford, MA, USA) with ClinMass Complete Kits (Immunosuppressants in whole blood, advanced–on-line analysis). The autosampler temperature was set at 10°C and the autosampler needle was washed with a strong needle wash solution of isopropanol:methanol:acetontitrile:H_2_O (1:1:1:1, v/v). A solution of 20% (v/v) methanol was used as weak needle wash. Analytes were ionized by electrospray ionization in the positive mode and detected on a triple quadrupole mass spectrometer (Xevo TQ-S, Waters, Milford, MA, USA). The capillary and the cone voltage were set at 3 kV and 40 V, respectively. The source offset was set at 60 V, the desolvation temperature at 400° C, the desolvation gas at 1000 L/h, the cone gas at 150 L/h, the nebulizer at 7 bar and the source temperature at 150° C. The instrument was controlled via MassLynx (version 4.1, Waters). Data were acquired, integrated and processed with TargetLynx (MassLynx v4.1).

### Correlation of tacrolimus and near-infrared dye *in vitro*

Bottoms of 2 mL tubes (Eppendorf, Hamburg, Germany) were excised and replaced with SnakeSkin Dialysis Tubing, 10K MWCO, 22 mm (Thermo Fisher Scientific, Waltham, MA, United States). Tubes were filled with 200μL TGMS-TAC-NIRD and randomly divided into two groups–control group (n = 3, gel submerged in 1 mL PBS) and experimental group (n = 3, gel submerged in 1 mL PBS containing 10 μL Lipase from *Thermomyces lanuginosus* solution, ≥100,000 U/g [Sigma]). Tubes were placed in 50 mL Falcon tubes (Thermo Fisher Scientific) containing 10 mL PBS and then mounted on a shaker under the following incubation conditions: 37° C, 5% CO_2_ and 95% relative humidity, 50 rotations/minute. At each time point, the whole reservoirs (10 ml) were replaced with fresh 10 ml PBS and 5ml was lyophilized for tacrolimus detection using HPLC and rest 5ml was used for IR dye detection. NIRD measurements were performed with LI-COR Odyssey imager and a 96 well plate, as previously described. The concentration of tacrolimus was determined in HPLC using a reverse phase C18 column (4.6 mm internal diameter x 250mm length, 5μm) and maintained at 40°C, and the eluents were monitored at 213 nm. The mobile phase (100% acetonitrile) was used at a flow rate of 0.9 ml/min, and the retention time for tacrolimus was 5.2 min.

### Statistical analyses

Statistical analyses were performed with Prism software (GraphPad Software Inc., La Jolla, CA, United States). Statistically significant data are presented as follows: * p < 0.05; ** p < 0.01; *** p < 0.001; and **** p < 0.0001. The statistical tests used are mentioned in the respective figure legends. Raw data of all figures is shown in S1 File.

## Results

### TGMS-TAC releases tacrolimus in response to inflammatory stimulus *in vivo*

**Fig 1:**
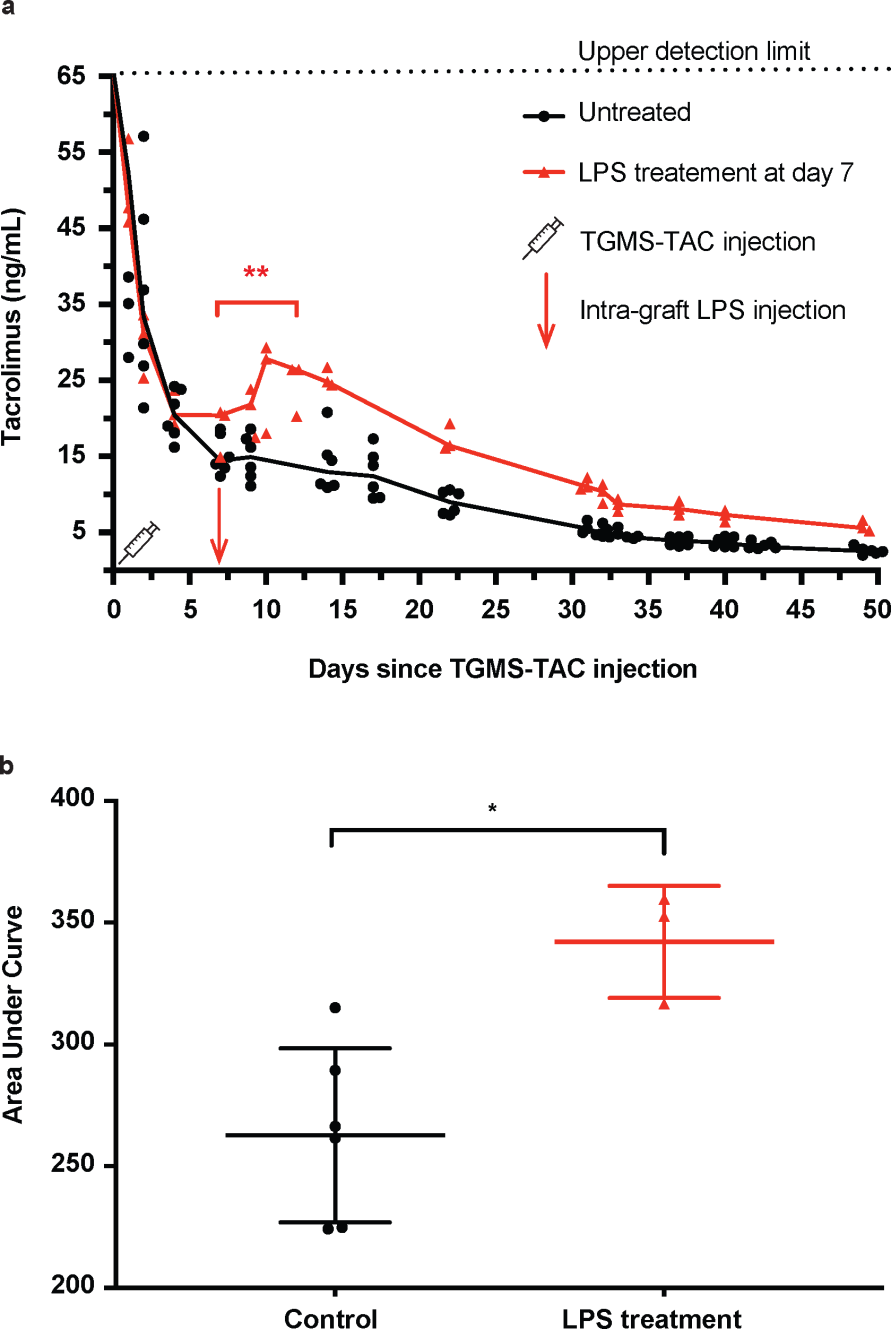
Tacrolimus release in blood of rats challenged or not with LPS. a) Longitudinal measurements of tacrolimus in blood of rats injected subcutaneously in the hind limb with TGMS-TAC. Increased tacrolimus levels after LPS challenge in the same limb (n = 3, red line), but no increase in unchallenged animals (n = 6, black line). Upper limit of detection of tacrolimus in blood by LC-MS/MS was 65 ng/mL, higher values are not reliably measurable. b) AUC analyses of untreated and LPS-treated animals (*P < 0.05, unpaired t-test).

Naïve Lewis rats receiving a subcutaneous injection of TGMS-TAC in the hind limb demonstrated an initial burst release of TAC, leading to a peak in blood TAC levels that remained above 25ng/mL for the first 72 h. This peak was followed by normalization to therapeutic levels for over a month and a subsequent drop to sub-therapeutic TAC levels that continued to be detectable for at least 50 days. In the group receiving a subcutaneous injection of LPS in proximity to the TGMS-TAC deposits on day 7, TAC levels were comparable for the first seven days. After LPS injection the animals demonstrated a significant increase of systemic TAC levels in the subsequent days reaching 18.7 ± 3.3 (mean ± SD) ng/mL TAC at day 7 and 24.4 ± 3.5 ng/ml at day 12 (p = 0.0076, one-way ANOVA with Tukey’s correction for multiple comparisons, Fig 1A). In addition, the area under the curve (AUC) of systemic TAC release was significantly higher in the LPS-treated animals compared to untreated animals (342.1 ± 13.3 ng/ml, n = 3 for LPS-treated versus 262.7 ± 14.6 ng/ml, n = 6 for untreated, p = 0.0108, unpaired t-test of AUC, Fig 1B).

**Fig 2:**
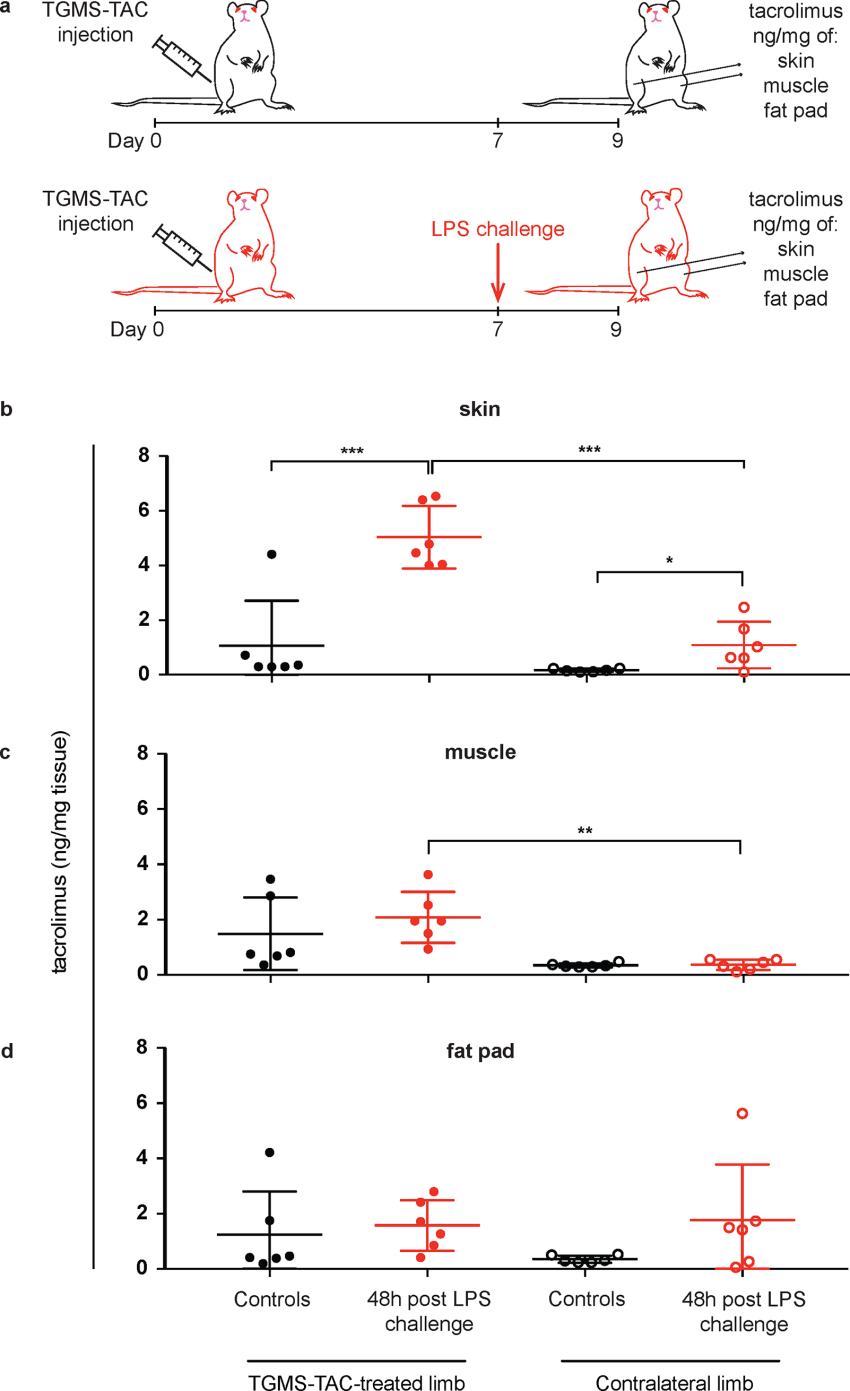
Tacrolimus levels in tissues of rats challenged or not with LPS. a) Experimental set-up. TGMS-TAC treated animals received or not a subcutaneous injection of lipopolysaccharides (LPS) 7 days after TGMS-TAC injection. 48 h after LPS challenge (or no challenge for controls), tissue levels of tacrolimus were measured by LC-MS/MS in b) skin, c) muscle and d) fat pad of TGMS-TAC treated and untreated, contralateral limbs (n = 6 rats per group). b)-d) Shown are individual data points for each animal, with indication of mean ± SD by lines. Paired t-tests were used for intra-group comparisons of same tissues; unpaired t-tests were used for inter-group comparisons of same tissues from either treated or contralateral limbs (* p < 0.05, ** p < 0.01, *** p < 0.001).

In order to measure local drug concentration, we performed an additional experiment in which animals were sacrificed 48 h after LPS challenge (or no challenge for control group, n = 6 per group) and muscle, skin and fat pad from treated and contralateral untreated limbs were collected for TAC measurements (Fig 2A). TAC skin levels of LPS-challenged animals were significantly higher in the treated limbs compared to the contralateral limbs (p = 0.0003, paired t-test, Fig 2B) and compared to the TAC skin levels in the treated limbs of the control group (p = 0.0007, unpaired t-test, Fig 2B). TAC skin levels in contralateral limbs of LPS-challenged animals were also significantly higher than the respective levels in the control group (p = 0.0254, unpaired t-test, Fig 2B). TAC levels in muscle of LPS-challenged animals were significantly higher in the limb receiving the LPS challenge in comparison to the contralateral limb (p = 0.0053, paired t-test, Fig 2C). TAC levels in the fat pad of LPS-challenged animals were comparable between limbs receiving the LPS challenge, and the contralateral limbs (p = 0.8134, paired t-test, Fig 2D). In the group without LPS challenge, there were no significant differences in TAC levels in skin, muscle, and fat pad between TGMS-TAC-treated limbs and untreated, contralateral limbs (p = 0.2442, p = 0.0771 and p = 0.2319, respectively, paired t-test). TAC skin levels of LPS-challenged limbs were significantly higher compared to the TAC levels in the underlying muscle and fat pad (p = 0.0173 and p = 0.0015, paired one-way ANOVA with Tukey’s post-hoc test).

### TGMS-TAC injection leads to foreign body reaction

**Fig 3:**
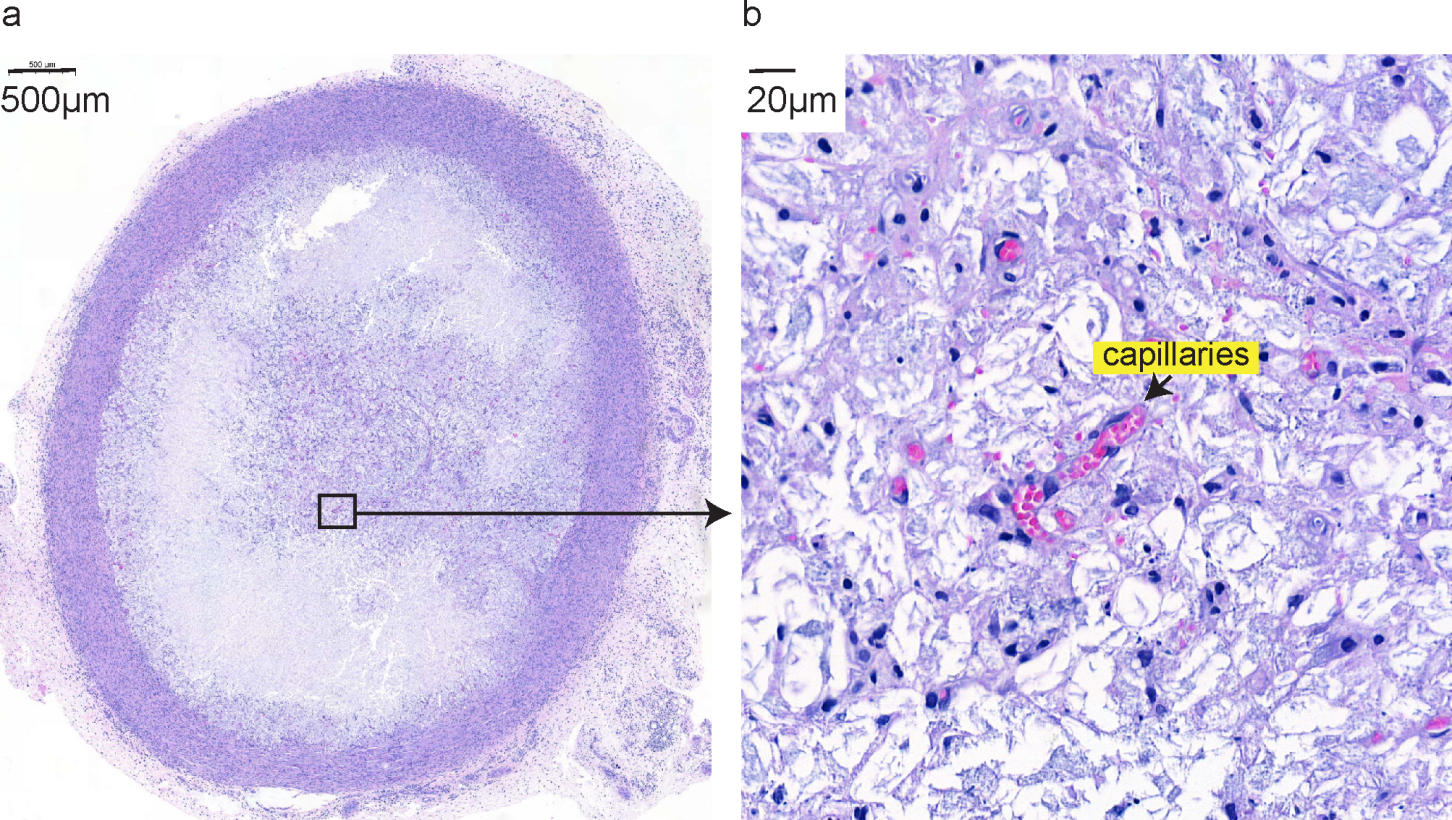
Hydrogel monitoring. Hydrogel deposits explanted for analyses on POD 9 show fibrotic capsule formation (a) and vascularization–capillaries formed inside the hydrogel deposit, indicated by an arrow (b). Shown are representative histological hematoxylin and eosin stained sections of hydrogel deposits at 3x (a) and 50x (b) magnification.

Explanted hydrogel deposits at day 9 after injection were analyzed histologically. All of them (n = 14) had perifocal “capsule” formation (Fig 3A), were granulomatous in 64.3% (9 animals) and were myofibroblastic in 57.1% (8 animals). Foreign body giant cells were noted in 35.7% of the cases (5 animals). Interestingly, despite capsule formation, the hydrogel was not isolated from its surroundings given the presence of capillaries with circulating erythrocytes deep within the gel (Fig 3B).

### Near-infrared dye release from TGMS-TAC-NIRD and correlation to tacrolimus release *in vitro*

**Fig 4:**
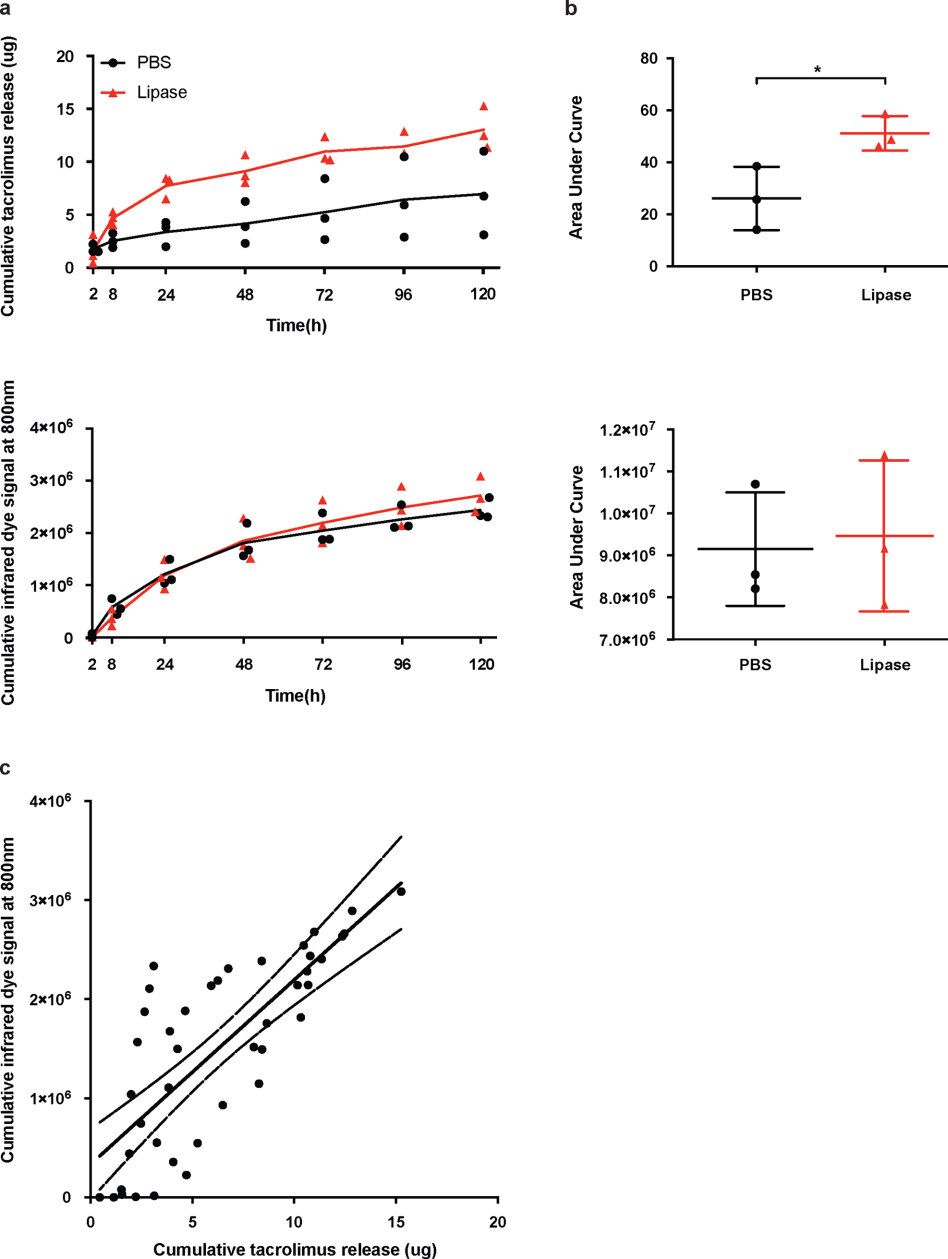
Tacrolimus and near-infrared dye release from TGMS-TAC hydrogel *in vitro*. a) Cumulative release of tacrolimus from TGMS-TAC under PBS or PBS spiked with lipase conditions over time and corresponding b) AUC analyses. c) Cumulative release of NIRD from TGMS-TAC under PBS or PBS spiked with lipase conditions over time and corresponding d) AUC analyses. e) Pearson correlation of tacrolimus and NIRD release from TGMS-TAC–linear regression with 95% confidence intervals.

Stability of the TGMS-TAC-NIRD hydrogel and correlation between drug and dye release was, at first, investigated in an *in vitro* assay. TGMS-TAC-NIRD were incubated with PBS only (n = 3), or PBS supplemented with lipase (n = 3). AUC analyses demonstrated that overall TAC release was significantly higher in lipase-spiked samples compared to controls (26.09 ± 7.05 ng/mL for control versus 51.21 ± 3.83 ng/mL for lipase, respectively, p = 0.0351, unpaired t-test of AUC, Fig 4A and 4B). However, the NIRD release from lipase-spiked samples was not significantly different compared to controls (unpaired t-test of AUC, Fig 4C and 4D). Correlation between the release of TAC and NIRD from the hydrogel was R^2^ = 0.6066 as computed by Pearson linear regression (Fig 4E).

### Near-infrared dye release from TGMS-TAC-NIRD and correlation to systemic and local tacrolimus levels *in vivo*

**Fig 5:**
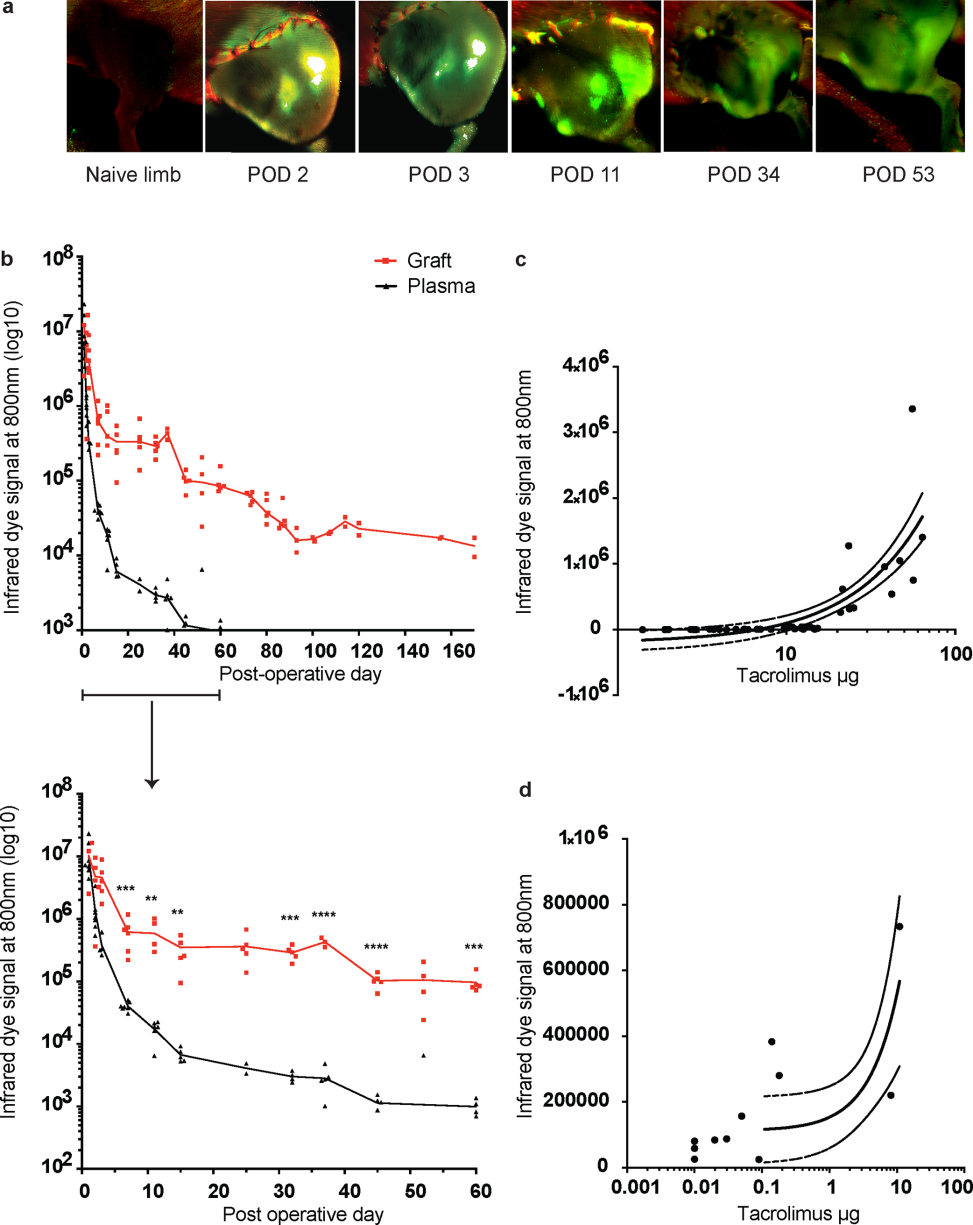
Near-infrared dye release from TGMS-TAC in a Brown Norway-to-Lewis rat hind limb transplantation model. a) Representative images of longitudinal monitoring of fluorescence emission at 800 nm from grafted limbs treated with NIRD-containing TGMS-TAC (n = 5). POD–Post-operative day; green–emission of NIRD at 800 nm; red–auto fluorescence of tissue, hair and sutures at 700 nm; white–overexposure. b) Longitudinal monitoring of fluorescence emission at 800 nm from grafted limbs and plasma (n = 5). Values are normalized for area, overexposed areas were excluded, and background subtraction was performed by imaging limbs and plasma before transplantation, and subtracting the mean obtained values. Featured is a close-up of the first 60 days, including analyses (** p < 0.01; *** p < 0.001; and **** p < 0.0001; multiple t-test with Holm-Sidak correction, p values are indicated directly above the corresponding time points). c) and d) Pearson correlation of tacrolimus and NIRD release from TGMS-TAC in plasma (c) and graft skin (d)–linear regression with 95% confidence intervals.

To assess the release kinetic of NIRD incorporated in TGMS-TAC *in vivo* and its potential value for hydrogel monitoring in VCA, we used a Brown Norway-to-Lewis rat hind limb transplantation model. Animals (n = 5) received four deposits of 250 μL TGMS-TAC-NIRD in the transplanted limb. Fluorescence emission at 800 nm from TGMS-TAC-NIRD into the surrounding graft tissue was monitored at selected time points (representative images in Fig 5A). Blood plasma was also collected at the same time points and imaged in a 96-wells plate to determine systemic NIRD release. Longitudinal analyses of plasma and graft fluorescence revealed that plasma fluorescence was no longer detectable after 60 days while intra-graft fluorescence persisted for more than 160 days (Fig 5B). Importantly, we observed rapid fluorescence signal decay in the peri-transplant periods (from day 0 to day 20 after transplantation) and a slower and more stable decrease in fluorescence in the subsequent period. Out of five grafts, three reached grade 3 of macroscopic rejection at post-operative days (POD) 94, 96, 96, and the remaining two were kept until the end of the study (POD 170) without any signs of rejection. To correlate NIRD emission with TAC levels locally in the graft and systemically in the blood, graft skin and peripheral blood were collected at selected time points for TAC measurements. Correlation of TAC and NIRD release from TGMS-TAC-NIRD in plasma was R^2^ = 0.6297 (Fig 5C), and in graft was R^2^ = 0.5619 (Fig 5D), as computed by Pearson linear regression.

## Discussion

We have developed TGMS-TAC, a hydrogel for localized immunosuppressive drug delivery directly into a transplant graft. Our findings in a rat VCA model suggest that TGMS-TAC is an efficient and safe alternative to systemic immunosuppression [8] Here we aimed at expanding our understanding and the clinical translatability of this therapeutic modality. Previously, we have shown *in vitro*, that TGMS-TAC acts in an on-demand manner, by releasing TAC in response to enzymes typically elevated during rejection-induced inflammation [7]. We now confirm this mechanism of action of TGMS-TAC *in vivo* in Lewis rats. Animals treated with TGMS-TAC and challenged with LPS–a potent inflammatory stimulus–near the TGMS-TAC deposit clearly demonstrated an increase in systemic blood TAC levels. This TAC-peak was not observed in control, unchallenged animals. Importantly, blood TAC levels in LPS-challenged animals were comparable to unchallenged animals prior to the LPS injection. Following the peak-release induced by the LPS challenge, TAC-levels remained slightly higher but followed the same trend of decrease as in the unchallenged animals. Consequently, more TAC was released from TGMS-TAC in LPS-challenged animals, as evidenced by higher AUC in comparison to the unchallenged group. In addition, tissue biopsies from the treated and the contralateral limbs revealed that TAC concentrations in skin and muscle in the LPS-treated limbs were significantly higher as compared to the respective levels in the contralateral, untreated limbs. In contrast, in the control animals’ tissue, TAC levels in treated and contralateral limbs were comparable. The skin TAC levels seemed to be most responsive to the LPS challenge, which can be explained by the fact that LPS was given subcutaneously. Both the LPS-treated and contralateral limb skin TAC levels were higher as compared to the respective TAC levels in the control group, further confirming that inflammation induce a consistent drug release form the hydrogel. These findings underline that TAC release from TGMS-TAC animals is highly dependent on the local inflammatory milieu.

As already observed in our first study [7], an initial burst release of TAC in blood was observed in the first 72 h after TGMS-TAC injection in naïve rats. Moreover, we observed tacrolimus release under non-inflammatory conditions *in vivo* that was not observed in our *in vitro* study (2). This may be due to inflammation triggered by hydrogel injection, to non-enzymatic hydrolysis of the hydrogel or to passive diffusion of non-encapsulated drug. Hydrogel deposits explanted at day 9 after injection consistently showed foreign-body reaction with formation of large capsules. We did not specifically investigate whether foreign body reaction was connected to local inflammation. Therefore, the possibility that the injection of the hydrogel can promote an initial inflammatory response at the injection-site deserves further investigation. Importantly, 50 days after injection, hydrogel deposits were almost completely resorbed, indicating that the capsule did not isolate the hydrogel from its surrounding, as also demonstrated by the appearance of capillary-like structure within the hydrogel. Other explanations for the burst and non-inflammatory release maybe a non-specific washout of TAC, which is not fully encapsulated by TGMS, or spontaneous (non-enzymatic) gel disassembly *in vivo*. Indeed, due to the modalities of hydrogel preparation, there might be some TAC and NIRD that is passively adsorbed. Since the gel preparation is done directly in the syringe, it is not possible to add washing step to remove these passively adsorbed drug or dye. Overall, our data demonstrate that after injection, the behavior of the hydrogel differs from what is demonstrated in vitro, requiring further investigations focused on reducing/controling foreign-body reaction, improving hydrogel preparation and drug encapsulation and/or modifying the hydrogel composition to reduce non-enzymatic hydrolysis of TGMS-TAC at the injection site.

In this paper we also assessed an optimization of TGMS-TAC by making it possible to be monitored non-invasively. To this aim we incorporated NIRD, which demonstrated a reasonable correlation with TAC release both *in vitro* and *in vivo*. An important observation, however, is that unlike TAC, NIRD was not selectively released in response to lipase from the hydrogel *in vitro*. A likely reason for this is that TAC is a highly lipophilic molecule, while the NIRD used in our study is a hydrophilic one. The hydrophilic NIRD could be passively released from the TGMS hydrogel in a non-enzymatic fashion, while hydrophobic TAC would preferentially remain within the hydrogel until it is released in the environment as a result of degradation of the hydrogel by lipase. Substituting the hydrophilic NIRD with a more hydrophobic one could be a potential way to solve this problem and improve the correlation between TAC and NIRD release from the hydrogel deposits. Ultimately, NIRD could serve as a potential surrogate marker for visualizing TGMS-TAC deposits *in situ* and non-invasively estimating the remaining amount of TAC.

A limitation of this study is that we investigated the TAC release from TGMS-TAC in the context of a local, acute inflammatory stimulus such as LPS. It remains to be elucidated whether inflammation at another anatomical site could influence the release of TAC from TGMS-TAC in a more “endocrine” fashion. This is a critical point, as the goal of TGMS-TAC is to provide relieved systemic immune depression in the context of environmental offenders, such as viruses, bacteria and fungi, as compared to systemic immunosuppression. On the other hand, systemic inflammation, for example caused by infection, is known to trigger graft rejection [18], and an increased local immunosuppression in the graft may protect the graft in a clinical setting.

Despite the limitations of our study we could demonstrate significant differences between TGMS-TAC behavior in inflammatory versus non-inflammatory conditions *in vitro* and *in vivo*. Moreover, we could visualize the hydrogel deposits *in situ*, allowing accurate estimation of the amount and distribution of TAC in the graft and in the blood. Further analyses that build upon our knowledge on TGMS-TAC could help bring this attractive localized inflammation-responsive drug delivery system closer to bedside.

## Supporting information

S1 File Raw data. (XLSX)
